# ‘Adult ADHD’ and ‘neurodevelopmental disorder’ – a critique of the latest socio-psychiatric ‘epidemic’

**DOI:** 10.1177/01410768241311321

**Published:** 2025-01-22

**Authors:** Derek Summerfield

**Affiliations:** Institute of Psychiatry at the Maudsley, London SE5 8AF, UK

The *British Medical Journal* notes that there is now an ‘unprecedented’ demand for services provided by the National Health Service for attention deficit hyperactivity disorder (ADHD).^1^ ADHD has been a diagnosis previously associated with children but the ‘epidemic’ now is of ‘adult ADHD’. This podium article examines this ‘epidemic’ in the light of three previous socio-psychiatric ‘epidemics’, all of which ended badly.

The first is multiple personality disorder (MPD), which was included as a diagnostic category in the Diagnostic and Statistical Manual (DSM) of the American Psychiatric Association in 1980. The Canadian philosopher Ian Hacking noted that only a handful of diagnosed cases had ever been made in the past but by 1986 MPD was epidemic, with over 6000 cases.^2^ Hacking noted that media interest helped public awareness of MPD to grow contagiously. Patients with MPD were seen as carrying part personalities – ‘alters’ – running in parallel, commonly up to 20 but some patients were judged by psychiatrists to be carrying hundreds of ‘alters’. But gradually criticism about over-diagnosis and the sheer dubiousness of the category mounted and the bubble burst. Psychiatrists stopped using MPD and it disappeared from the DSM, to be replaced by dissociative identity disorder.

Second, the ‘recovered memory’ debacle in the 1990s. Urged on by psychotherapists who believed that the transcending cause of adult distress or disturbance was childhood sexual abuse, even if not remembered, women began to accuse family members, particularly fathers. Families splintered, and criminal trials and long jail sentences ensued for some innocent men before the bubble burst as the weight of clinical opinion eventually came to judge ‘recovered’ memories as false memories, a product of suggestibility. Much harm had been done.^3^

A third sobering and recent example of a socio-psychiatric bubble that burst concerns the gender identity furore, one where evolving public attitudes and claims regarding gender became problematically intertwined with the professional output of the clinic. Gender dysphoria was a recognised category in the DSM 5th edition. The Tavistock gender identity clinic, the national service, had 210 referrals in 2010–2011 but 5000 in 2020–2021. This ballooning cohort of referrals, children and adolescents, were largely birth gender women whereas birth gender men had predominated in the past. After often perfunctory assessment, a proportion of these ended up on puberty blockers and went on to have surgery, such as breast removal. Unease among parents and some clinicians eventually forced a re-think, following High Court involvement and the Keira Bell case. The Cass report criticised the lack of evidence surrounding benefits and the risks associated with puberty blockers.^4^ There are now restrictions on the use of puberty blockers and the Tavistock gender clinic has closed.

The three examples above describe the sudden rise of a psychiatric or psychological category and attendant treatment field, initially under-scrutinised by academics and clinicians, becoming briefly an epidemic fact of public life before collapsing with harm done. Will this be the fate of the new epidemic regarding ‘adult ADHD’? One spur has been that the DSM has widened the diagnostic criteria, making it easier to qualify. This (and also a category like autism) are now being referred to as ‘neurodevelopmental’ disorders – though there have been no defining brain findings to underpin the biological validation that formally distinguishes a ‘disease’ (like Alzheimer’s) from a ‘syndrome’ (like ADHD, autism, depression, PTSD, bipolar disorder, schizophrenia and virtually all clinical psychiatry). The psychiatric profession has never reminded the general public that these psychiatric categories are merely symptom clusters with indistinct borders, not clustered by nature but by the DSM committees. Lacking proven biological anchoring, syndromal categories are susceptible to cultural trends regarding over-medicalisation and societal contagion. Reference to ‘neurodevelopmental disorders’ is thus ungrounded, an advertising label promoting a new field and essentially disease-mongering.

We are witnessing a spectacular rise from nothing in the societal profile of ‘adult ADHD’, in part fuelled by self-administered checklists. The same checklists would qualify people for other diagnoses – or for none at all – but ‘adult ADHD’ is the diagnosis of the day. Once something is declared real, it becomes real in its consequences. Many people seem relieved to acquire a social identity predicated on the diagnosis, not least the moral shift that may accompany this.

Amphetamine prescription rates are surging: for example, prescriptions for adults aged 30–34 years have increased by 146% in three years.^1^ Regarding evidence base, Castells et al. searched CENTRAL, MEDLINE, Embase, PsychINFO, 10 other databases and two trials registers. They assessed these studies as of ‘low, or very low quality’. They also noted death rates attributed to prescribed amphetamines.^5^ Amphetamines have a long history of abuse and addiction, including acute psychosis and a schizophrenia-like picture with chronic usage. I predict another bubble bursting in due course and an unhappy ending to the ‘adult ADHD’ story.

The chairman of the DSM 4 task force Dr Allen Frances publicly expressed regret for what he recognised as three false ‘epidemics’ spurred on by its publication: ADHD, autism and childhood bipolar disorder. He became a critic of the expanding boundaries of psychiatry and of the medicalisation of normal human behaviour, like over-diagnosis and over-treatment of the ‘worried well’. In his seminal dissection of the health industry, ‘Medical Nemesis’ (1975), Ivan Illich concluded that the more our lives were medicalised, the sicker we felt.^6^ People seek a diagnosis as the explanation for the problems of living yet life simply does not break down into currently fashionable psychiatric categories. The media take the ADHD story literally, as if ADHD had always been a clear-cut condition ‘out there’ but only now fully recognised, separable from the person carrying it, like TB or cancer. The real epidemic out there is the epidemic of ADHD diagnosing and prescribing.

Lastly, the four ‘epidemics’ related above suggest that a major factor in what exacerbates and prolongs over-medicalisation, with its attendant costs, is the reluctance of the mental health field to critique itself until it is too late. Dr Allen Frances’s warnings stand.

## Declarations

### Guarantor

DS.

### Provenance

Commissioned; peer-reviewed by Aileen O'Brien.
